# Quantification of Calcium Amount in a New Experimental Model: A Comparison between Ultrasound and Computed Tomography

**DOI:** 10.1371/journal.pone.0148904

**Published:** 2016-02-09

**Authors:** Kris Gillis, Gezim Bala, Bram Roosens, Isabel Remory, Sophie Hernot, Steven Droogmans, Bernard Cosyns

**Affiliations:** 1 In vivo Cellular and Molecular Imaging (ICMI) laboratory, Faculty of Medicine and Pharmacy, Vrije Universiteit Brussel (VUB), Brussels, Belgium; 2 Centrum voor Hart- en Vaatziekten (CHVZ), Department of Cardiology, Universitair Ziekenhuis Brussel (UZ Brussel), Brussel, Belgium; Brigham and Women's Hospital, Harvard Medical School, UNITED STATES

## Abstract

**Purpose:**

Calcification is an important prognostic factor in aortic valve stenosis. However, there is no ultrasound (US) method available to accurately quantify calcification in this setting to date. We aimed to validate a new US method for measuring the amount of calcium in an *in vitro* model, and compare it to computed tomography (CT), the current imaging gold standard.

**Materials and Methods:**

An agar phantom (2% agar) was made, containing 9 different amounts of calcium-hydroxyapatite Ca_5_(PO_4_)_3_OH (2 to 50mg). The phantoms were imaged with micro-CT and US (10 MHz probe). The calcium area (area_calcium_) and its maximum pixel value (PV_max_) were obtained. These values were summed to calculate CT and US calcium scores (∑(*area*_*calcium*_ × *PV*_*max*_)) and volumes (∑*area*_*calcium*_). Both US- and CT-calcium scores were compared with the calcium amounts, and with each other.

**Results:**

Both calcium scores correlated significantly with the calcium amount (R^2^ = 0.9788, p<0.0001 and R^2^ = 0.8154, p<0.0001 for CT and US respectively). Furthermore, there was a significant correlation between US and CT for calcium volumes (R^2^ = 0.7392, *p*<0.0001) and scores (R^2^ = 0.7391, *p*<0.0001).

**Conclusion:**

We developed a new US method that accurately quantifies the amount of calcium in an *in vitro* model. Moreover it is strongly correlated with CT.

## Introduction

Calcific aortic valve stenosis (AVS) is the most frequent valvular heart disease and leading cause for valvular surgery in Western countries.[1] In this setting the amount of calcification has important clinical implications. It is a powerful predictor of morbidity and mortality in calcific aortic valve disease (CAVD)[2,3] and allows a more accurate assessment of the disease severity in low-flow low-gradient AVS[4]. In therapeutic decision making, calcification burden and localization are also important during pre-operative evaluation for aortic valve (AV) replacement or bicuspid leaflet repair.[5] In patients, treated with trans-catheter aortic valve implantation, the amount and localization of calcium is associated with an increased risk for paravalvular regurgitation.[6] As CAVD develops, the presence of calcification has an important prognostic value and is associated with faster progression of AVS.[3,7] Recently, calcification has been suggested as a target for new treatment in patients[8], and in experimental settings serial examinations allowed to evaluate the effect of treatment on CAVD[9]. Therefore, imaging modalities are mandatory, not only for prognostic assessment but also for serial follow-up with monitoring of progression and potential regression of CAVD.

Computed tomography (CT), currently the reference technique for quantification of AV calcifications, is less suitable for repetitive evaluations, due to irradiation.[2] Therefore, new techniques for precise quantification of calcifications are needed, that permit serial follow-up of CAVD patients.

Echocardiography is primarily used to evaluate AV morphology and function, but previous studies demonstrated that ultrasound (US) can also be used to assess the severity of cardiac calcifications.[10–12] However these semi-quantitative scoring systems were only obtained visually and the exact amount of calcium could not be determined. Other US methods, such as intravascular ultrasound (IVUS), are already used in atherosclerosis to characterize plaque calcification, but can not be applied to moving structures such as the AV.[13] Finally, integrated backscatter (IB), the integrated average energy of US backscattered reflections, could be used to extract information concerning valvular tissue composition and to detect AV calcifications in rats.[14] IB was recently validated for the detection of progression and regression of AV calcifications *in vivo*, as well as for the differentiation between AV thickening and AV calcifications in rats.[14,15] However, to date neither ultrasound technique, using gray-scale or IB analysis, has been validated to quantify the precise amount of calcium, which currently can only be measured with CT.[2]

In the present study we aimed and achieved to validate a new US method for quantifying the amount of calcium in an *in vitro* model and to compare the results obtained with CT.

## Materials and Methods

### Calcium-Containing Agar Phantom

A 2% agar-solution was made by dissolving 4mg agar powder (Select Agar®, Invitrogen^TM^, ThermoFisher Scientific) in 200ml demineralized H_2_0 at 160°C. After one hour of continuously stirring on a hot magnetic stir plate, the temperature was decreased to 80°C and the agar-solution slowly stirred further until all bubbles were removed.

Nine plastic bars, each 15x3x20mm, were glued together with interspaces of 1 cm to form one block. This block was suspended, hanging from a tripod, with the tip of each plastic bar located at 10mm above the bottom of a recipient of 100x100x25mm, and the agar solution was poured in. The agar was cooled down for 45 minutes at room temperature. The plastic bars were then removed, leaving an agar mold with 9 rectangular holes of 15x3x5mm. In each hole, a different amount of dry Ca_5_(PO_4_)_3_OH powder (Hydroxyapatite type II 20μm, Bio-Rad Laboratories) was homogenously spread (2mg, 4mg, 6mg, 8mg, 10mg, 20mg, 30mg, 40mg and 50mg). Finally, the remaining agar was poured on top until a phantom thickness of 25mm was reached. The phantom was cooled down for hardening overnight at room temperature.

### Ultrasound Imaging

**Fig 1:**
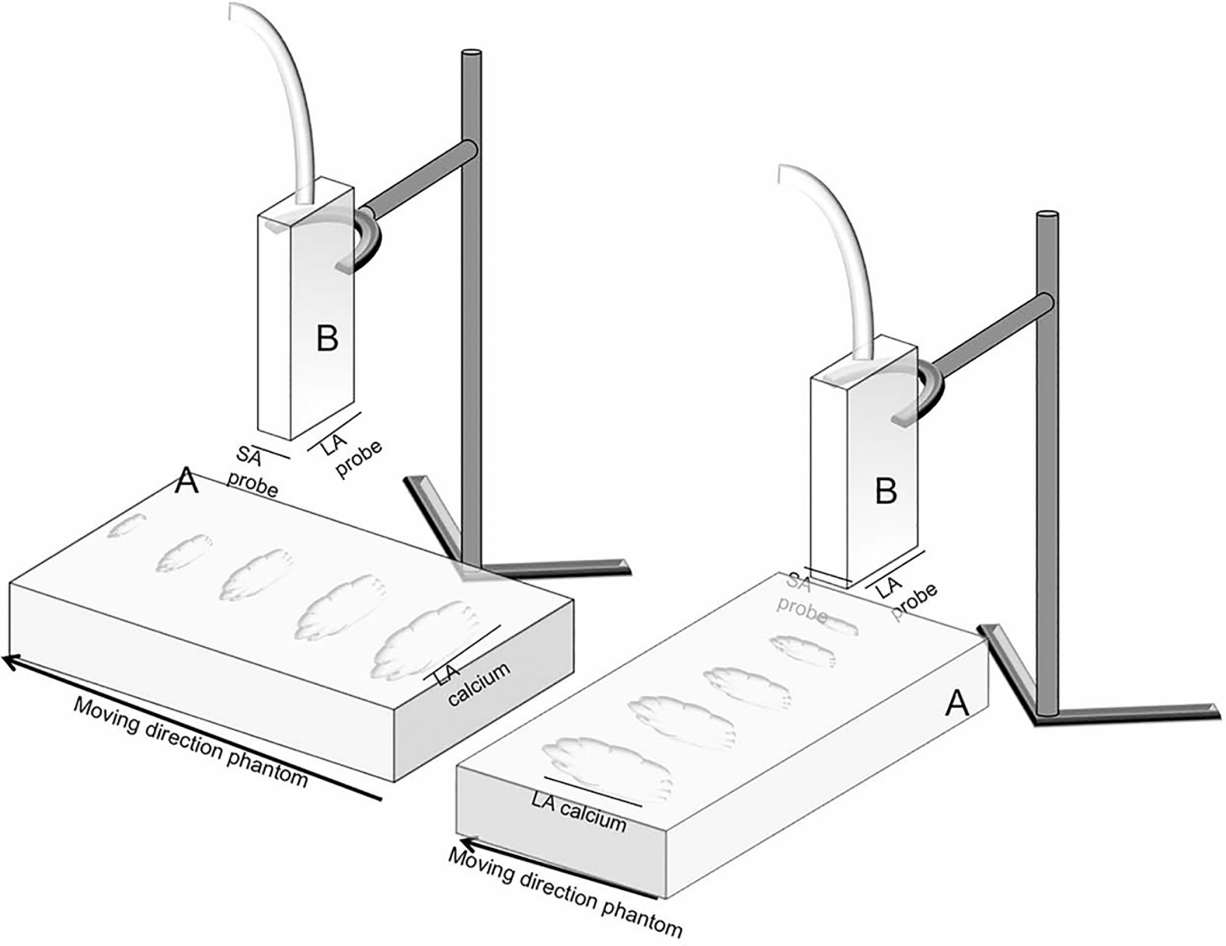
Schematic overview of the position of phantom and ultrasound probe during ultrasound image acquisition. Schematic overview of the position of phantom and ultrasound probe during ultrasound image acquisition. Left. Position to obtain long axis images. The long axis of the probe (LA probe) is parallel to the long axis of the calcium (LA calcium). The phantom moves in direction along the short axis of the probe (SA probe). A = agar phantom with 5 increasing amounts of calcium, B = ultrasound probe mounted on support stand. Right. Position to obtain short axis images. The long axis of the probe (LA probe) is perpendicular to the long axis of the calcium (LA calcium). The phantom moves in direction along the short axis of the probe (SA probe). A = agar phantom with 5 increasing amounts of calcium, B = ultrasound probe mounted on support stand.

The phantom was insonified with a Vivid 7 Pro system and 10S neonatal transducer (GE Medical Systems, Milwaukee, WI, USA). Settings were kept constant, with frame rate 204.1 FPS, frequency 11,5MHz and focus position at the center of the calcium spot. The US probe was fixed on a support stand, in direct contact with the agar. First, images were made with the long axis of the probe parallel to the long axis of the calcium spot. (Fig 1) The phantom was moved along the short axis of the probe, and every millimeter an image was stored for offline analysis. This resulted in a stack of long axis images, covering the total volume of each calcium spot. The same procedure was repeated with the long axis of the probe perpendicular to the long axis of the calcium spot to obtain a stack of short axis images. (Fig 1)

### Ultrasound Calcium Scoring

#### US calcium scoring

For every calcium mass, US analysis was performed offline with OsiriX software(OsiriX 3.6.1, 64-bit, Pixmeo, Geneva, Switzerland). The Dicom images were first converted from red-green-blue to gray-scale to enable pixel intensity analysis. Then, in each image belonging to a calcium speck, a wide ROI was placed around the calcium. Using OsiriX plugin “set pixels” the pixel value (PV) outside this ROI was set to zero. The pixel value ranged from 0 to 250. A threshold of 130 PV was assigned for calcium selection. This threshold was based on the maximum gray-scale density of agar, was able to exclude agar, and to include all calcium pixels. Within the ROI, all pixels with *PV* ≥ 130 were selected as calcium using the plugin “global thresholding”. The area (area_calcium_) and maximum intensity (PV_max_) of the selected pixels were automatically provided.

A calcium score was calculated for each image, by multiplying area_calcium_ by PV_max_. The total calcium score for the whole calcium spot was obtained by the summation of all the calcium scores in all images belonging to that spot. This was done for the short axis and long axis images, and the mean of both results was defined as the “US calcium score”. The US calcium volume was calculated by the summation of the area_calcium_. All analyses were performed randomly, blinded for the calcium amount.

#### cIB calcium scoring

For every calcium mass, IB analysis was performed offline with EchoPac PC software (version 110.0.0, GE Medical Systems, Milwaukee, Wi, USA) in a random order and blinded for the calcium amount. In each image the IB value (IB_calcium_), in decibels, was measured in an ellipsoidal ROI delineating the calcium spot. The area of this ROI was calculated:

$${area}_{calcium}=\left(\frac{longest\;diameter\;ROI}{2}\;\times \;\frac{shortest\;diameter\;ROI}{2}\right)\;\pi$$

For calibration, the IB_agar_ value was measured in a circle-shaped ROI of 1mm diameter, 10mm above the calcium. The calibrated IB value (cIB) of each image was calculated: *cIB* = (*IB*_*calcium*_ − *IB*_*agar*_) × *area*_*calcium*_. The short axis cIB score and the long axis cIB score were obtained by summation of the cIB values of short and long axis images respectively. Finally, the “cIB calcium score” was obtained by the mean of the short and long axis cIB values.

### Micro-CT Imaging and Calcium Scoring

Micro-CT (SkyScan 1178, SkyScan/Bruker microCT, Kontich, Belgium) imaging of the phantom was performed using two digital X-ray cameras which scanned over 180° at a resolution of 83μm, a rotation step of 1.08°, 50 kV, 615 μA and 121s image acquisition time. The reconstruction was performed using a modified Feldkamp cone-beam algorithm (NRecon, SkyScan/Bruker microCT, Kontich, Belgium) to return a stack of 2D cross-sectional images. These images were converted into Dicom format in the medical image data analysis tool Amide (Amide1.0.4 64-bit, copr. Andreas Loening). The phantom was scanned with water and air as reference values (0 and -1000HU respectively) for conversion of PV into Hounsfield Units (HU). The pixel values ranged from 0 to 65535.

OsiriX was used to calculate the calcium volume and CT calcium score for each calcium mass. Therefore, in the first and last image of a calcium spot, a wide ROI was drawn around the calcium, and the missing ROIs in every image in between were automatically generated.

All pixels outside these ROIs were set to PV zero. A threshold of 700HU, which corresponds with denser bone and hydroxyapatite, was assigned for calcium selection.[16,17] This threshold was also based on the agar density, and was set high enough to exclude agar. The HU threshold was converted into PV threshold, based on the following equation:

$${HU}_{threshold}=\frac{\left({PV}_{threshold}-{PV}_{water}\right)}{\left({PV}_{water}-{PV}_{air}\right)}\;\times \;1000$$

Within each ROI, calcium was selected by applying the PV threshold, which corresponded with 700HU. All pixels above or equal to the PV threshold were extracted and the area_calcium_ and PV_max_ were automatically provided. The PV_max_ was converted into HU (HU_max_).

In analogy with the Agatston score, a weighting factor (wf) was assigned according to the maximal density (HU_max_) in the ROI. Based on the PV threshold and on micro-CT settings, we assigned the wf as follows, wf = 0 for < 700HU, 1 for 700-999HU, 2 for 1000-1999HU, 3 for 2000-2999HU and 4 for > 3000HU. The CT calcification score (CCS) was obtained by multiplying wf by area_calcium_. The CCS of every calcification in all the images of a calcium spot was summed to obtain the final “CT calcium score”.

Furthermore, the CT calcium volume was calculated through summation of the area_calcium_ of all images of a calcium speck. All analyses were performed randomly, blinded for the amount of calcium.

### Statistics

Correlations between imaging techniques were evaluated by the Pearson correlation coefficient. Receiver-operator curves (ROC curves) of both imaging techniques were calculated to evaluate their sensitivity to the amount of calcium. The intraclass correlation coefficient (ICC) with 95% confidence interval (95%CI) was calculated for intra-observer and inter-study variability. All p values were calculated two-tailed. A p value < 0.05 was considered significant.

## Results

### Correlation of the Calcium Amount with US Calcium Score and CT Calcium Score

**Fig 2:**
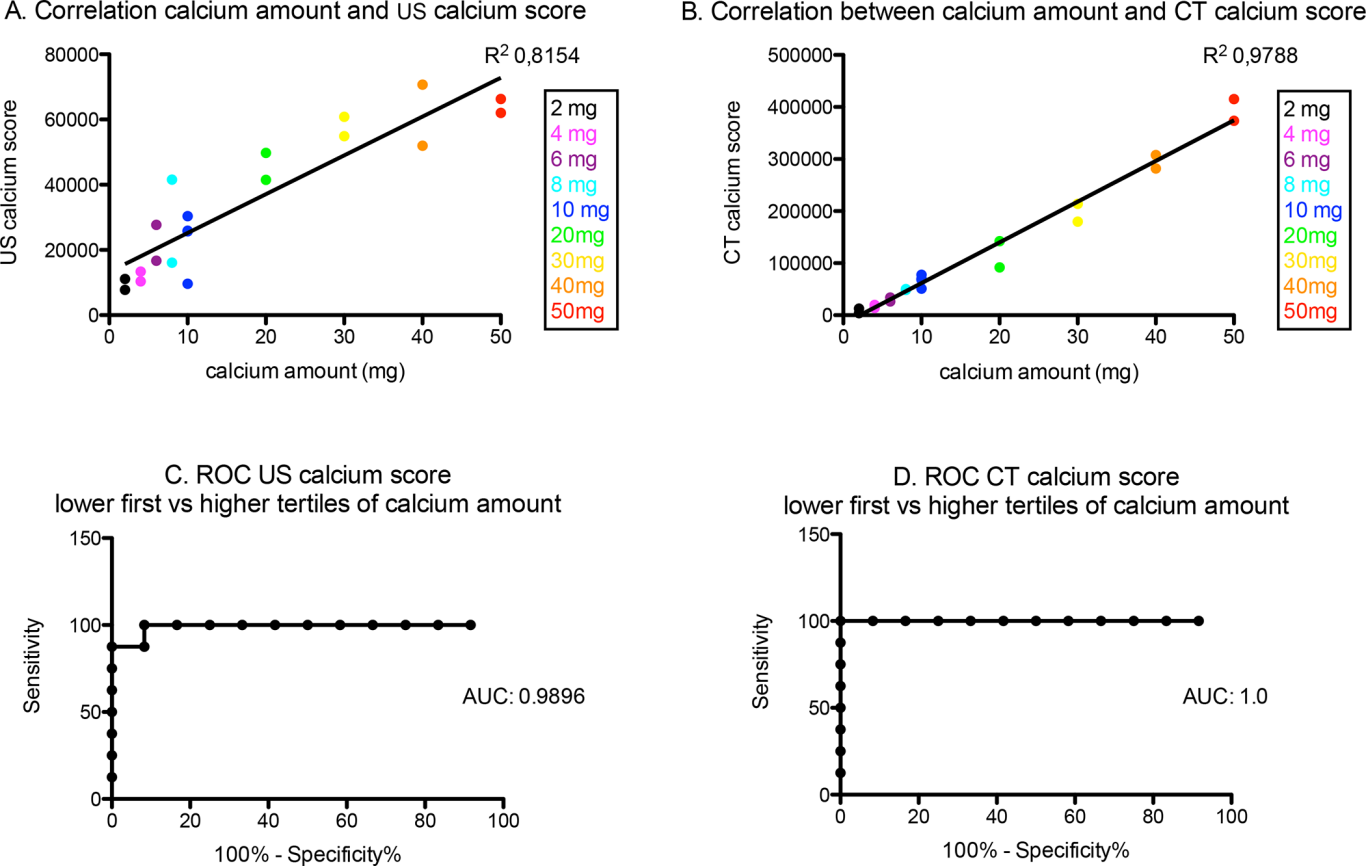
Correlation of the calcium amount with US calcium score and CT calcium score. (A) The correlation of the calcium amount with the US calcium score. There is a significant correlation between the amount of calcium (2-50mg) and the US calcium score, with R^2^ = 0.8154, p<0.0001. (B) The correlation of the calcium amount with the CT calcium score. There is a significant correlation between the amount of calcium (2-50mg) and the CT calcium score, with R^2^ = 0.9788, p<0.0001. (C). The ROC curve of US calcium score. The ROC curve of the US calcium score, for distinguishing between the lower tertile and the higher tertiles of calcium amount. AUC = 0.9896 with SD +/-0.06178; p = 0.0002906. ROC = Receiver-operator curve. AUC = Area under the curve. (D) The ROC curve of CT calcium score. The ROC curve of the CT calcium score, for distinguishing between the lower tertile and the higher tertiles of calcium amount. AUC = 1.0 with SD +/-0.0; p = 0.0002151. ROC = Receiver-operator curve. AUC = Area under the curve.

The US calcium score had a very good correlation with the calcium amount, with R^2^ = 0.8154, p<0.0001. (Fig 2A) The mean US calcium score ranged from 9416 (SD = 2375) for 2mg calcium to 64170 (SD = 13258) for 50mg calcium.

There was also a significant correlation between CT calcium score and the amount of calcium, with R^2^ = 0.9788, p<0.0001. (Fig 2B) The mean CT calcium score ranged from 8358 (SD = 6027) for 2mg calcium to 394224 (SD = 29476) for 50mg calcium.

The ROC curve was calculated to distinguish the lower tertile of calcium amount (2 to 10mg) from the higher tertiles (20-50mg), and showed an area under the ROC curve (AUC) of 0.9896 +/- 0.06178SD, with p = 0.0002906. (Fig 2C) A cutoff US calcium score of 35951 was calculated which was able to detect a calcium amount above 10mg with 100% sensitivity and 91.67% specificity.

The ROC curve was also calculated for the CT calcium score to distinguish the lower tertile from the higher tertiles of calcium amount, and showed an AUC of 1.0, with p = 0.0002151. (Fig 2D) A cutoff CT calcium score of 84893 was calculated, which was able to detect a calcium amount above 10mg with 100% sensitivity and 100% specificity.

Both US calcium score and CT calcium score are also very accurate for detection of very low amounts of calcium with threshold 4mg (cut-off US calcium score 12243, sensitivity 88.89%, specificity 100%, AUC 0.9444; and cut-off CT calcium score 13190, sensitivity 100%, specificity 100%, AUC 1.0), and very high calcium amounts with threshold 40mg (cut-off US calcium score 50886, sensitivity 100%, specificity 87.50%, AUC 0.9688; and cut-off CT calcium score 247938, sensitivity 100%, specificity 100%, AUC 1.0)

### Correlation between US Calcium Score and CT Calcium Score

**Fig 3:**
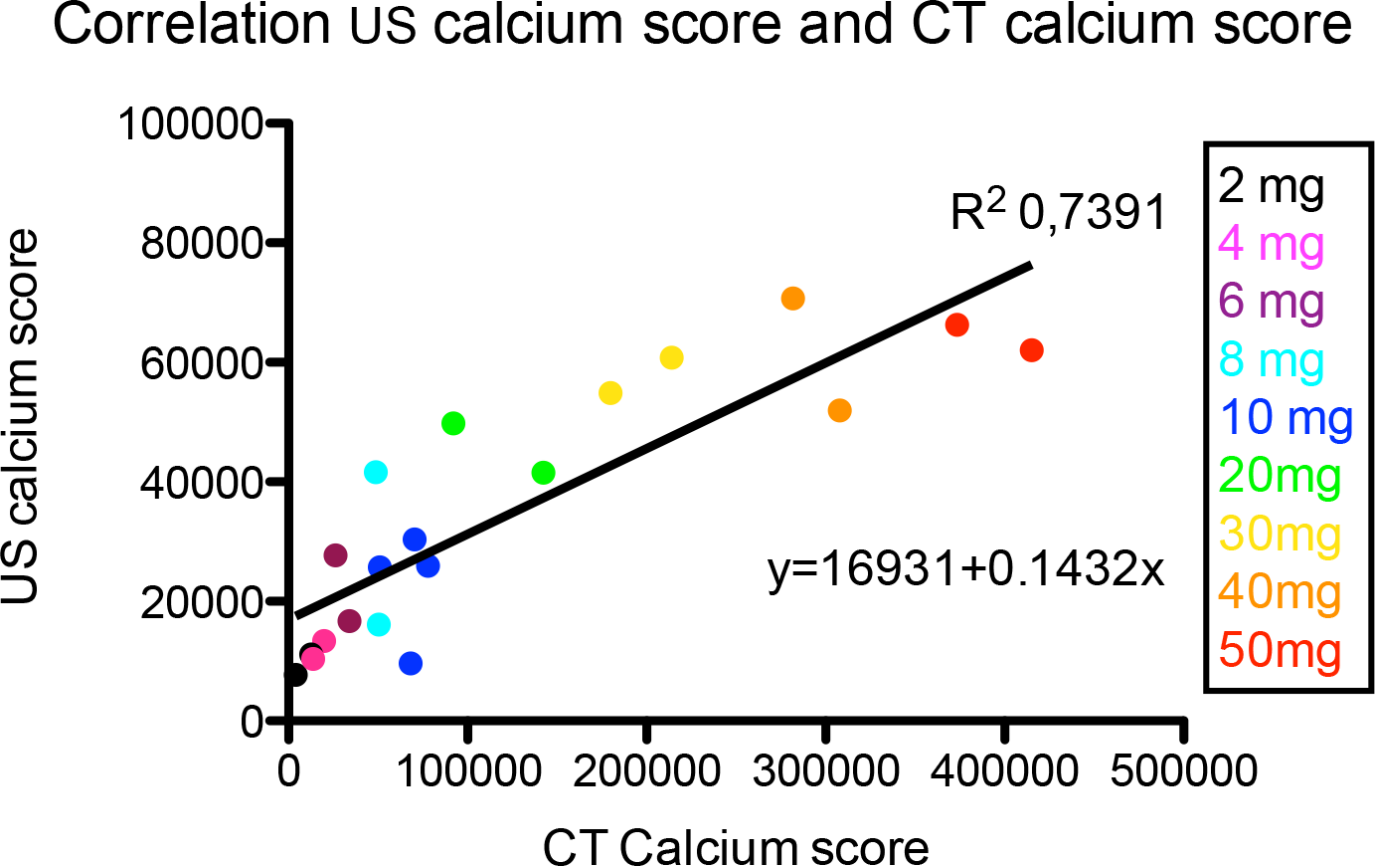
The correlation between US calcium scores and CT calcium score. The correlation between calcium scores measured with CT (CT calcium score) and ultrasound (US calcium score) for different amounts of calcium (2-50mg). There is a significant correlation between the two techniques with R^2^ = 0.7391, p <0.0001.

There was a strong correlation between the calcium scores measured by CT and US analysis for the different amounts of calcium, with R^2^ = 0.7391, p <0.0001. (Fig 3) The comparison of both ROC curves (Fig 2C and 2D) showed no significant difference (p = 0.535) between the two techniques for distinguishing a low amount from the higher amounts of calcium.

### Correlation of Calcium Amount with US Calcium Volume and CT Calcium Volume

**Fig 4:**
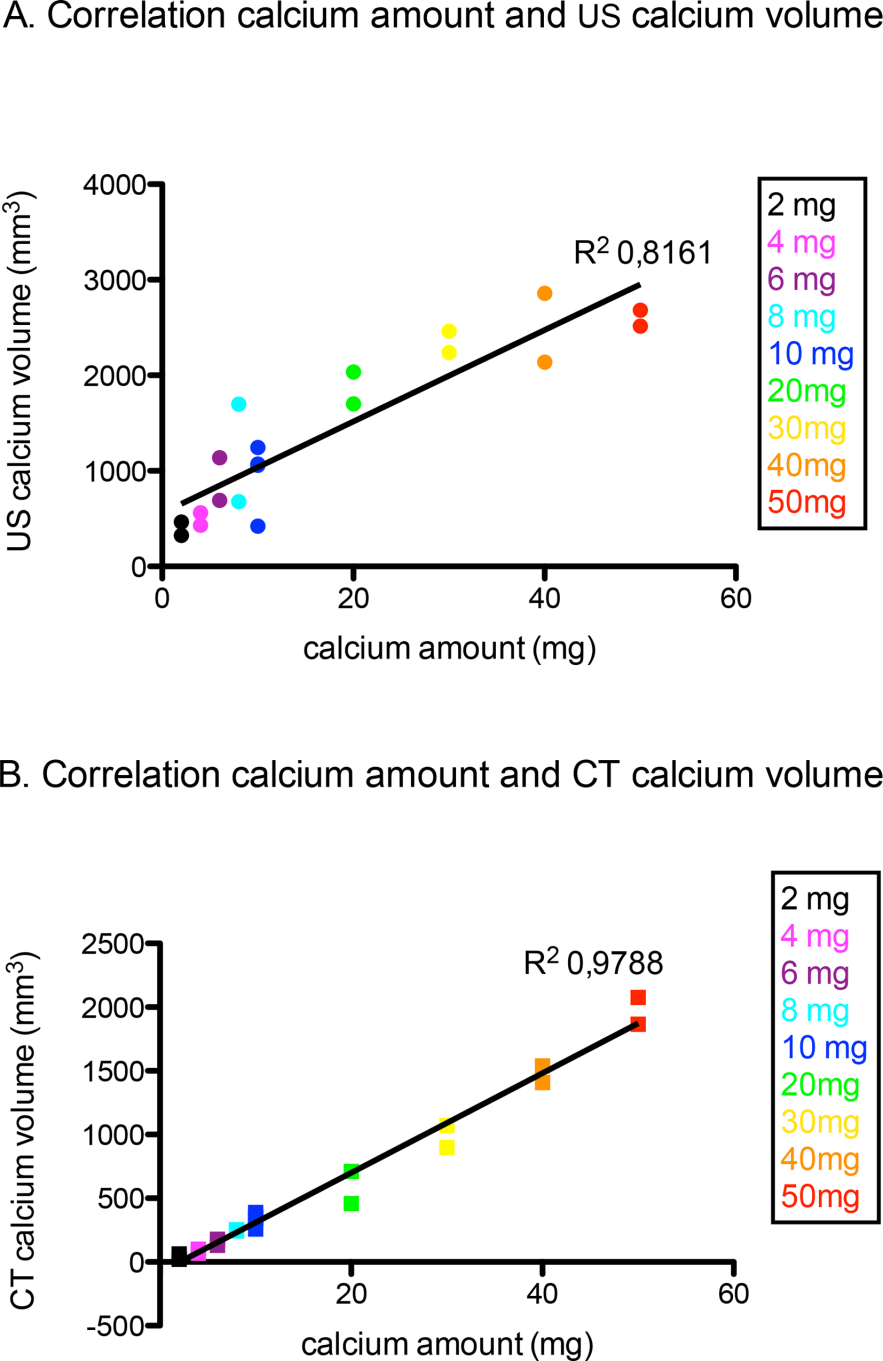
Correlation of calcium amount with US calcium volume and CT calcium volume. (A) The correlation between the calcium amount and the US calcium volume. There is a significant correlation between the amount of calcium (2-50mg) and the US calcium volume, with R^2^ = 0.8161, p<0.0001. (B) The correlation between the calcium amount and the CT calcium. There is a significant correlation between the amount of calcium (2-50mg) and the CT calcium volume, with R^2^ = 0.9788, p<0.0001.

There was an excellent correlation between the calcium volume measured with CT or US and the actual amount of calcium. The US calcium volume correlated significantly with the calcium mass, with R^2^ = 0.8161, p<0.0001 (Fig 4A), and CT calcium volume also correlated well with the calcium amount with R^2^ = 0.9788, p<0.0001. (Fig 4B)

### Correlation between US Calcium Volume and CT Calcium Volume

**Fig 5:**
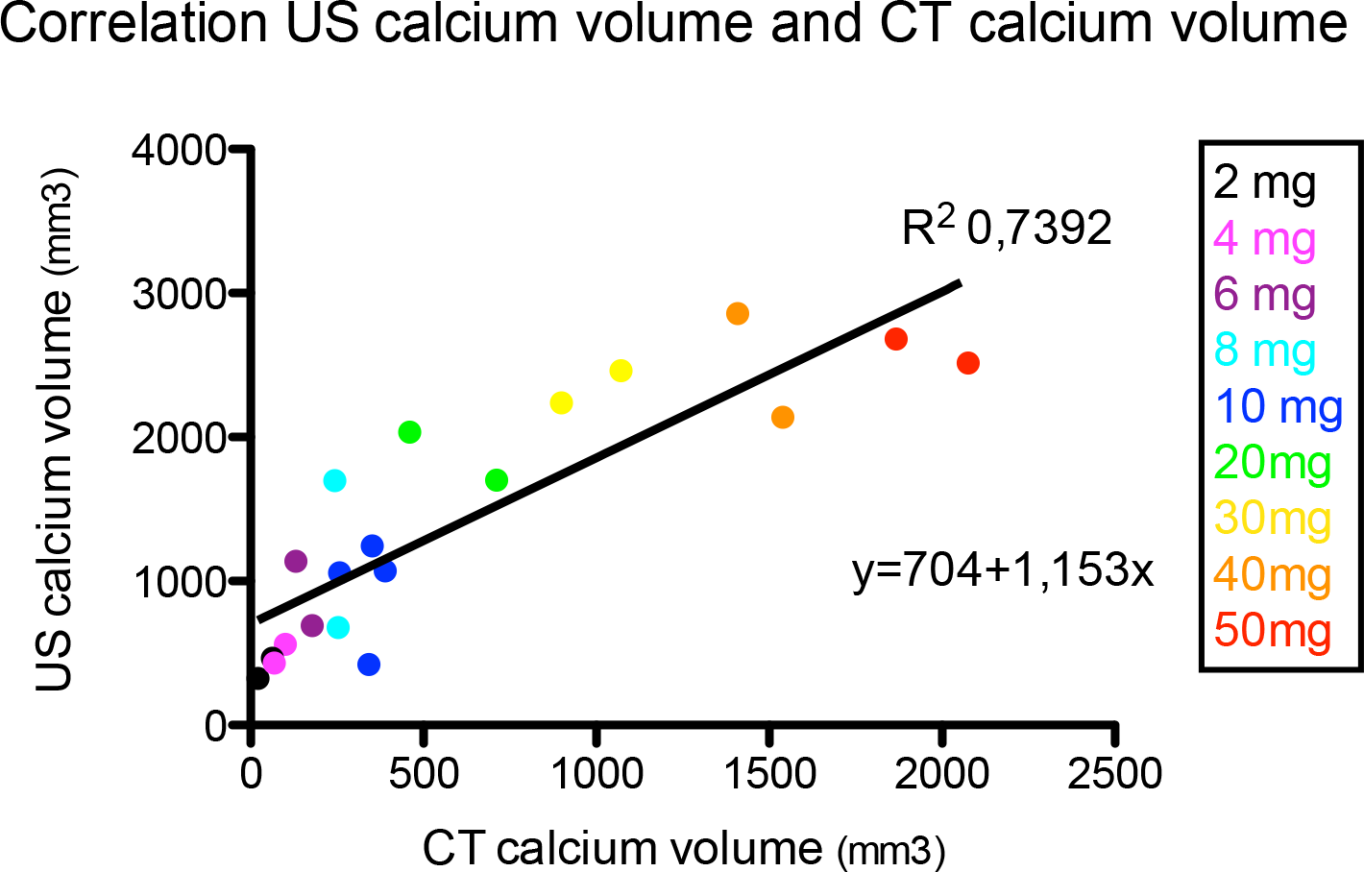
The correlation between the US calcium volume and CT calcium volume. The correlation between the calcium volume measured with CT and ultrasound, for different amounts of calcium (2-50mg). There is a significant correlation between the two techniques with R^2^ = 0.7392, p<0.0001.

There was a significant correlation between both imaging modalities for the measurement of the calcium volume, with R^2^ = 0.7392, p<0.0001. The volumes calculated by US were systematically higher than those calculated by CT. (Fig 5)

### Correlation between US Calcium Score and cIB Calcium Score

**Fig 6:**
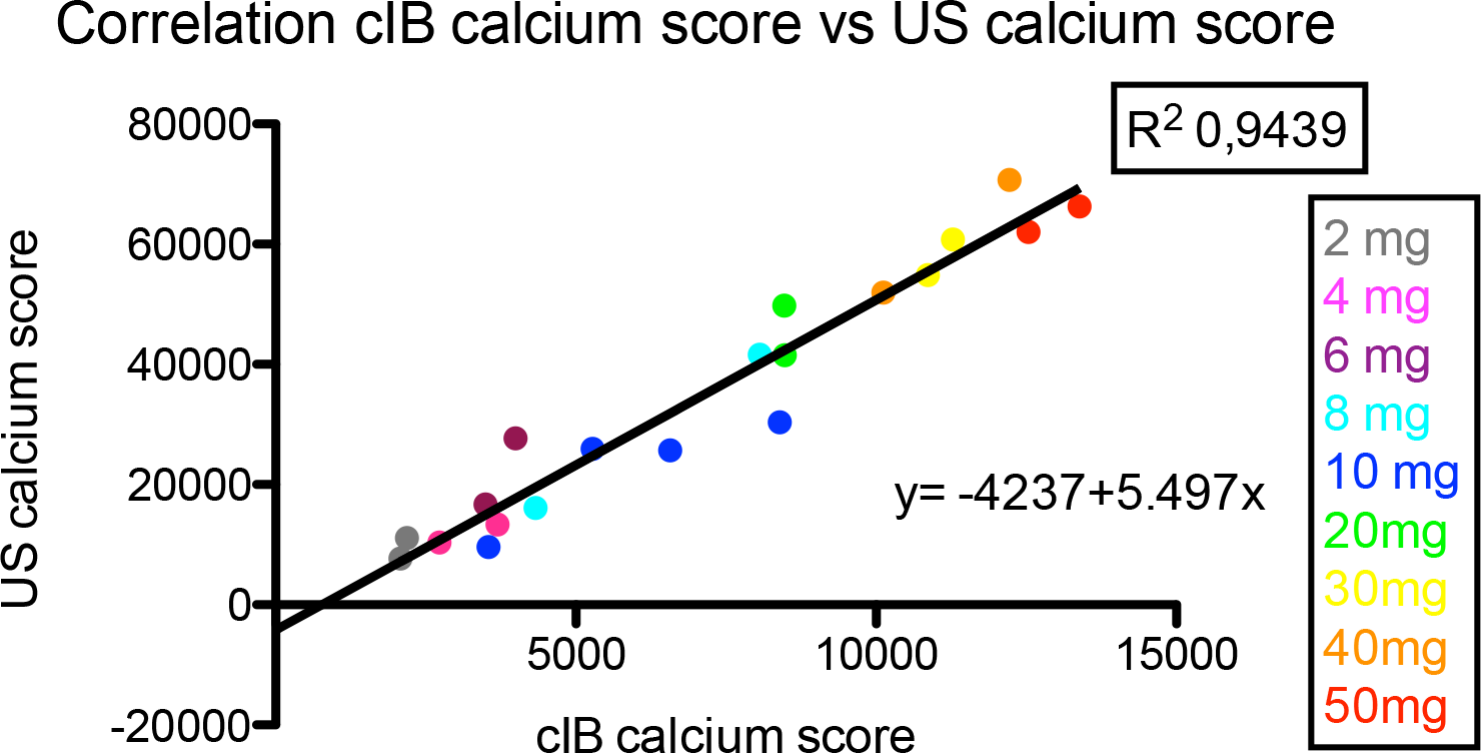
The correlation between cIB calcium score and US calcium score. The correlation between calcium scores measured with cIB (cIB calcium score) and ultrasound (US calcium score) for different amounts of calcium (2-50mg). There is a significant correlation between the two techniques with R^2^ = 0.9439, p <0.0001.

We found a very strong correlation between the calcium scores measured by US gray-scale and cIB analysis for the different calcium amounts, with R^2^ = 0.9439, p <0.0001. (Fig 6)

### Reproducibility

For the US calcium score and US calcium volume measurements, we found a good intra-observer variability (ICC = 0.997, with 95%CI 0.956–0.999 and ICC = 0.997 with 95%CI 0.956–0.999, respectively). We also found a good inter-study reproducibility for both US calcium score and volume in the same phantom (ICC = 0.959, with 95%CI 0.719–0.996 and ICC = 0.996 with 95%CI 0.738–0.996, respectively) as well as between different phantoms (ICC = 0.857, with 95%CI 0.526–0.962 and ICC = 0.851 with 95%CI 0.510–0.961, respectively).

## Discussion

Using US, we developed a new semi-automatic method to accurately quantify the amount of calcium in an *in vitro* model. We were able to calculate US scores that correlate with the various amounts of calcium present in a phantom. Furthermore, the US calcium score was compared with the CT calcium score, showing a strong correlation between the two imaging modalities.

Phantoms are an important tool for the evaluation and validation of imaging modalities. Agar, with its acoustic velocity, density and impedance similar to human tissue, has previously been used as phantom material for US and CT.[18,19] In earlier studies, agar phantoms were made to mimic cardiac tissue.[20] We therefore developed an agar-based phantom, compatible with both imaging modalities, and easy to imbed calcium in. This *in vitro* phantom-based model allowed us to insert exact amounts of calcium, in order to validate US as a quantification technique and to compare it with CT.

CAVD is an active disease process involving endothelial damage, inflammation and calcification with hydroxyapatite deposition.[21–23] To mimic these calcifications more closely, we developed a phantom containing hydroxyapatite. Hydroxyapatite is a stable calcium salt, present in heavily calcified valves and in valves with minor calcifications.[24–26] Multiple other forms of calcium can be detected in calcified cardiovascular tissue, such as octacalciumphosphate, beta tricalciumphosphate and dicalciumphosphate, and amorphous calcium phosphate, which is suggested to play a role as precursor for hydroxyapatite.[27–30] However, the exact composition of calcific deposits is not known and may change during the calcification process. It is therefore theoretically impossible to develop a phantom, identical to the *in vivo* situation. We used hydroxyapatite, the final and stable calcium salt, that specifically can be detected and which contribution in calcific deposits is therefore more clearly established.[22,29] Moreover, hydroxyapatite is detected by CT during the evaluation of valvular calcifications, which makes it the ideal calcium salt to be used for the comparison of US and CT calcium quantification.[4,6,31] Nevertheless, when the US calcium score is evaluated in a clinical setting, the potential influence of other calcium salts should be taken into account. As to this *in vitro* study, we are confident that, although our model does not reflect the complexity of the clinical situation, it is a first and important step in the application of US for the quantification of aortic valve calcifications.

### Calcium Quantification

With this *in vitro* model we could detect and quantify the amount of calcium using US gray-scale analysis, through the widely available imaging software OsiriX. Previous studies have validated IB for the detection of progression and regression of AV calcifications, and for differentiation between AV thickening and calcifications in rats.[9,15,32] Therefore, we compared the gray-scale analysis with cIB analysis, and found a strong correlation between the two techniques. Since the software for IB analysis is less widely available and does not allow a semi-automatic approach, this may limit its feasibility and potentially cause a high observer-dependency. We favored the OsiriX US calcium score because this software allowed semi-automatic observer-independent rapid calcium detection, was more accessible and would consequently be more convenient for clinical practice.

A previous study suggested that real-time visual assessment of AV calcification with a semi- quantitative score was superior to gray-scale analysis of still frames, compared to palpation and visual surgical evaluation of the AV calcification. However, no histological nor CT calcium measurement was objectively performed.[33] In our study, we therefore sought to develop a robust and objective US method for calcium quantification and compare it to CT, a technique that is known to quantify calcium in a reliable and reproducible way. By using both short and long axis frames, instead of using one still frame, we obtained a more reliable assessment of the calcification. This approach should be further evaluated in clinical practice.

Calcium detection was obtained by selecting all pixels above a 130PV threshold. This value was based on the maximum gray-scale density of agar. To avoid a potential effect of US settings on gray-scale distribution, we kept all settings constant during image acquisition, allowing the use of the same threshold throughout the study. The obtained US calcium score correlated very well with the amount of calcium. The linear correlation and the wide range of US calcium scores (from 9416 (SD = 2375) for 2mg to 64170 (SD = 13258) for 50mg calcium) permit to easily derive and differentiate different amounts of calcium starting from the US calcium score. Furthermore the US calcium score was reproducible and therefore potentially interesting for calcium quantification in clinical practice. However, we observed that at higher calcium amounts, above 40mg, the US calcium score moves slightly towards a plateau. This may indicate a calcium saturation phenomenon. It may also partially be explained by an increasing effect of acoustic shadowing, which can make deeper parts of a big calcium spot more difficult to evaluate. Based on a study of calcifications on bioprosthetic valves, however, calcium amounts more than 40mg seem very high and are unlikely to occur in clinical practice.[34] Although at these extreme amounts of calcium the US calcium score might underestimate the calcium extent, further studies are mandatory to elucidate its clinical relevance. Furthermore, we calculated the US calcium volume, which correlated well with the calcium amount. The determination of volumes and localization of calcium could be of interest for preoperative evaluation.

To obtain our US calcium score, we used the maximal density in the ROI, similar to the CT Agatston score.[2] This approach however, may have the potential disadvantage not to take into account the effect of spreading of calcium. Further studies are needed to investigate the effect of calcium spreading on both scoring systems and to explore the use of the mean density in the ROI as a way to overcome these potential effects.

CT, currently the reference technique for imaging valvular calcifications, uses the valvular Agatston score, primarily designed for coronary arteries and based on a 130HU threshold for calcium detection.[2] However, in our study we used micro-CT, and a threshold of 130HU was too low, leading to selection of agar density. Based on the agar, water and air densities and normal HU range for calcifications, we selected a threshold of 700HU which corresponds with denser bone and hydroxyapatite.[16,17] This threshold permitted to calculate a CT calcium score, similar to the Agatston score, with a very good correlation with the actual calcium amount and with our US calcium score. Absolute values of both techniques are not comparable, because CT and US comprise different pixel density scales. However there is a significant correlation between the techniques, suggesting that US calcium score is a valuable method to quantify calcium, compared to CT. Moreover, the ROC analysis showed that US calcium score is a very sensitive score to distinguish low amounts of calcium from higher amounts of calcium. When compared to the ROC curve of the CT calcium score, there is no significant difference (p = 0.535) between the two techniques, suggesting that the US and CT calcium score have a comparable sensitivity and specificity for calcium quantification.

We also found a very good correlation between US and CT calcium volume calculations. However in absolute values, US calcium volume is systematically higher, which may indicate a lower threshold for calcium detection with US compared to CT.

### Clinical Translation

Because this study involves an *in vitro*, phantom-based model, translation into clinical practice may comprise some difficulties. First, a phantom is a static model where in clinical practice moving structures may influence the analysis. We fixed the probe on a support stand and kept the angle of insonification constant, perpendicular to the phantom, while in clinical practice angle-dependent scattering may occur and alter the US calcium score. Furthermore we obtained good image quality by bringing the probe in direct contact with the agar, which is a good ultrasound conductor. In clinical practice application of ultrasound gel is necessary, which may have an impact on the ultrasound transmission and scattering. Nevertheless, as previous clinical studies showed good results with visual US calcium scoring, our semi-automatic objective technique should not be significantly altered by the insonification angle or the presence of ultrasound gel.[11,12] Secondly, variable US equipment settings may interfere with the calcium scoring, making retrospective analysis of patient databases potentially challenging. As in our study, settings should be standardized and kept constant when performing prospective investigations. CT however, faces the same difficulties. Thirdly, we used a 10MHz US probe and high frame rate, to obtain high quality images in our small phantom. In clinical practice other probes, with frequencies ranging from 1 to 5 MHz for transthoracic or 2 to 7 MHz for transesophageal echocardiography, are often used. The difference in frequency and frame rate may have an impact on the US calcium score. This makes extrapolation of our absolute values and threshold potentially difficult. Further studies are necessary to investigate the effect of different US angles, settings and probes on the US calcium score analysis. The development of our phantom could contribute to explore these effects in a standardized way. As transesophageal probes emit higher frequencies than transthoracic probes, it should be interesting to investigate the use of both US modalities for calcium scoring in clinical practice.

Furthermore, differences exist between US and CT in terms of resolution, which may influence the accuracy of calcium quantification. The resolution of US depends mainly on the transducer and a high frequency and focused US beam will provide a better axial and lateral resolution. A 2 to 5MHz transthoracic US probe has an axial resolution of about 1.2 to 0.47mm.[35] The CT scans used in clinical practice have a resolution of 0.25–0.5 mm, which is better than the 5MHz probes.[36] However, when US frequency is increased, for example by using transesophageal echocardiography of 7MHz, the spatial resolution can be improved significantly to 0.33mm. Clinical studies therefore should be performed to emphasize the use of both transthoracic and transesophageal echocardiography to quantify AV calcifications. At the other hand, the temporal resolution of US (5ms) is much better than CT (70-160ms), which makes ultrasound very suited for the evaluation of moving structures such as the aortic valve.[37]

Variable attenuation of US *in vivo* may also limit the use of our absolute values for calcium identification. This effect should be explored in further studies. Nevertheless, in an individual patient, attenuation of US is expected to remain the same over time, which could make our US calcium score suited for serial follow up of individual patients.

A potential clinical limitation would be the patient’s echogenicity where a poor acoustic window could cause insufficient echocardiographic image quality. In this subpopulation, CT should remain the technique of choice. On the contrary, CT acquisition may be limited in certain patients (i.e arrhythmias) and US calcium score may represent a valuable alternative in these cases. One additional advantage of US calcification scoring, apart from the lack of ionization, is the possibility to integrate this measurement into any comprehensive echocardiographic examination for AV evaluation, increasing the versatility of echocardiography even more.

### Conclusions

In this study we developed a new semi-automatic ultrasound method and calculated an US calcium score that accurately quantifies various amounts of calcium in an *in vitro* model, and that correlates very strongly with the CT calcium score.

## Supporting Information

S1 File Underlying data1. Raw data phantom 1a cIB 10-50mg.
(XLSX)

S2 File Underlying data2. Raw data phantom 1a CT 10-50mg.
(XLSX)

S3 File Underlying data3. Raw data phantom 1a. US 10-50mg.
(XLSX)

S4 File Underlying data4. Raw data phantom 1b. cIB 10-50mg.
(XLSX)

S5 File Underlying data5. Raw data phantom 1b. CT 10-50mg.
(XLSX)

S6 File Underlying data6. Raw data phantom 1b. US 10-50mg.
(XLSX)

S7 File Underlying data7. Raw data phantom 2a. cIB 2-10mg.
(XLSX)

S8 File Underlying data8. Raw data phantom 2a. CT 2-10mg.
(XLSX)

S9 File Underlying data9. Raw data phantom 2a. US 2-10mg.
(XLSX)

S10 File Underlying data10. Raw data phantom 2b. cIB 10-50mg.
(XLSX)

S11 File Underlying data11. Raw data phantom 2b. CT 2-10mg.
(XLSX)

S12 File Underlying data12. Raw data phantom 2b. US 2-10mg.
(XLSX)

S13 File Underlying data13. raw data reproducibility interstudy. between phantoms 1b vs 3a. US 10-50mg.
(XLSX)

S14 File Underlying data14. Raw data reproducibility interstudy. between phantoms 2b vs 3b. US 2-10mg.
(XLSX)

S15 File Underlying data15. raw data reproducibility interstudy. same phantom 3a vs 3a. US 10-50mg.
(XLSX)

S16 File Underlying data16. Raw data reproducibility intraobserver. phantom 1a. US 10-50mg.
(XLSX)

S17 File Underlying data17. Raw data reproducibility intraobserver. phantom 2a. US 2-10mg.
(XLSX)
